# A Modular Cloning System for Standardized Assembly of Multigene Constructs

**DOI:** 10.1371/journal.pone.0016765

**Published:** 2011-02-18

**Authors:** Ernst Weber, Carola Engler, Ramona Gruetzner, Stefan Werner, Sylvestre Marillonnet

**Affiliations:** Icon Genetics GmbH, Halle/Saale, Germany; Virginia Tech, United States of America

## Abstract

The field of synthetic biology promises to revolutionize biotechnology through the design of organisms with novel phenotypes useful for medicine, agriculture and industry. However, a limiting factor is the ability of current methods to assemble complex DNA molecules encoding multiple genetic elements in various predefined arrangements. We present here a hierarchical modular cloning system that allows the creation at will and with high efficiency of any eukaryotic multigene construct, starting from libraries of defined and validated basic modules containing regulatory and coding sequences. This system is based on the ability of type IIS restriction enzymes to assemble multiple DNA fragments in a defined linear order. We constructed a 33 kb DNA molecule containing 11 transcription units made from 44 individual basic modules in only three successive cloning steps. This modular cloning (MoClo) system can be readily automated and will be extremely useful for applications such as gene stacking and metabolic engineering.

## Introduction

Synthetic biology promises to revolutionize biotechnology by engineering organisms with novel phenotypes not found in nature. Applications include the microbial production of chemical precursors, novel antibiotics and biofuels [1], the creation of synthetic attenuated viruses for use as vaccines [2] and the engineering of a minimal free living cell [3].

An element essential for synthetic biology is the ability to physically assemble complex DNA molecules containing large numbers of natural or artificial genetic elements. Impressive progress has been achieved in the past few years with the development of methods that allow assembly of large pieces of DNA of up to the size of entire bacterial genomes [4], [5], [6], [7], [8]. The majority of these methods is based on the use of homologous recombination (both *in vivo* and *in vitro*), which provides independence from the presence of any restriction sites in the fragments to assemble. Generation of organisms with novel phenotypes will however not only require the ability to assemble large pieces of DNA, but will also need methods that allow generation of many construct variants for optimization of a desired phenotype. Indeed, since a desired phenotype cannot be predicted directly from gene sequences only, development of strains and optimization of phenotypes will require the ability to generate multiple combinations of various coding sequences as well as many variants of their regulatory sequences. Such optimization does not necessarily need to operate at genome scale, and in fact, work currently done for metabolic engineering already belongs to this type of effort. In this context, what is needed are methods that allow generation of constructs or construct libraries containing enough genes for pathway engineering, i.e. in the size range of 10 to 100 kb. Despite considerable work done in metabolic engineering in the past few years, methods currently used for construct assembly are still limiting, as most of the work is still performed using standard DNA construction techniques that require extensive planning and multiple cloning steps.

To make such work more efficient, it is however useful to view DNA construction not just as a process for assembly of raw pieces of DNA, but rather as a process that allows assembly of discrete functional genetic elements. Since synthetic biology can be viewed as a form of engineering, it should be able to learn from existing mature technologies such as mechanical engineering. An essential factor for fast and reliable engineering of complex devices is standardization of their basic parts. In the case of synthetic biology, standardization would allow to reuse previously validated genetic elements from one application to the next, and allow the free exchange between different users. It should also allow the development of standardized construct assembly strategies that would help simplify the planning of cloning strategies and minimize the number of cloning steps required to obtain a desired construct.

The first attempt to standardize DNA construction, NOMAD, was made 15 years ago [9]. The authors proposed that libraries of modules of defined structure could be built and shared by the community. NOMAD modules are flanked by sites for the restriction enzyme StyI, which make them compatible with a specifically designed destination vector. Modules can be combined together in any order, but are cloned sequentially one module at a time to form a composite module, which can then be further subcloned. A major step forward was the development of the BioBrick standard, which allows assembly of constructs from basic biologic parts such as promoter, ribosome binding site and terminator [10]. Assembly of two basic BioBrick parts results in a composite part that has the same structure as the basic parts in terms of flanking restriction sites (the basic and composite parts are idempotent). This feature allows the same procedure to be repeated again to obtain larger constructs. Since the first BioBrick standard, various standards and assembly protocols have been developed in order to optimize the sequence junctions between parts or make cloning more efficient [11], [12]. However, both NOMAD and the various BioBrick standards are limited in their ability to assemble multiple DNA fragments in a single step, and still rely on procedures that limit their potential for automation such as extraction of DNA fragments from gels or the requirement for the design of custom primers for specific constructs [13].

We present here a modular and hierarchical cloning system that allows any desired eukaryotic multigene construct to be assembled from sets of pre-made standardized genetic modules, including promoters, 5′ untranslated regions, signal peptides, coding sequences and terminators. This cloning system is based on the Golden Gate cloning technology, a method that allows highly efficient directional assembly of multiple DNA fragments in a single reaction [14]. In order to prove the general feasibility of this modular cloning system and to show its potential, a 33 kb construct encoding 11 transcription units (made from 44 individual basic modules) was generated in only three successive one-pot cloning steps.

## Results

### Technological background and general considerations

The principle of Golden Gate cloning is based on the special ability of type IIS restriction enzymes to cleave outside of their recognition site [15]. When these recognition sites are placed to the far 5′ and 3′ end of any DNA fragment in inverse orientation, they are removed in the cleavage process, allowing two DNA fragments flanked by compatible sequence overhangs to be ligated seamlessly. Since type IIS restriction sites can be designed to create different overhangs, which are referred to as fusion sites from here on, directional assembly of multiple fragments is feasible [16]. For assembly, all DNA fragments can be simply provided as uncut plasmids, and are combined with the destination vector, T4 DNA ligase and the type IIS restriction enzyme in a single reaction mix. The use of restriction-ligation allows the assembly of multiple fragments with extremely high efficiency: we have shown earlier that up to 10 DNA fragments can be assembled with 95-100% of colonies obtained containing the expected construct [14].

**Figure 1:**
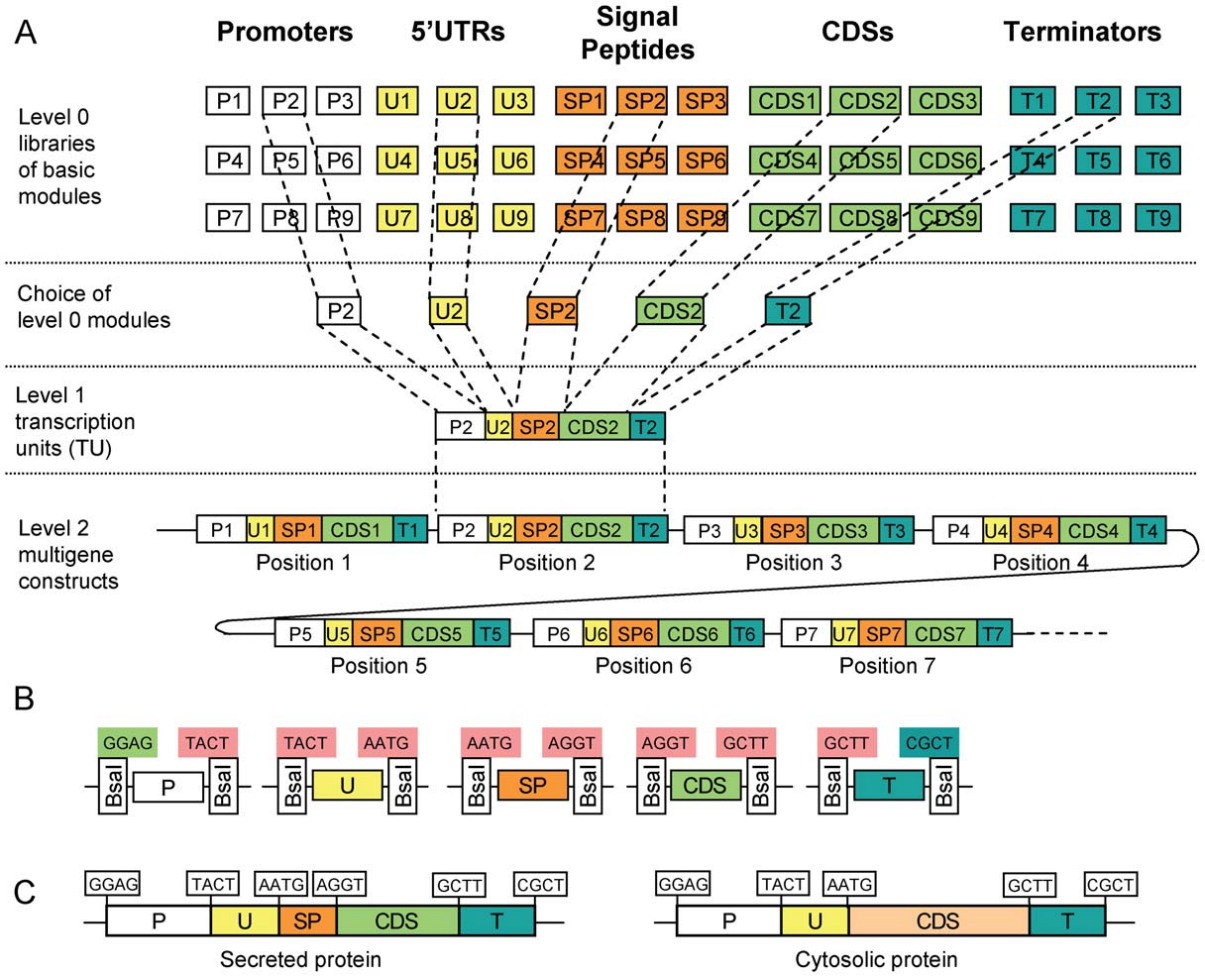
General overview of the hierarchical and modular cloning system. (**A**) Libraries of basic (level 0) modules contain cloned and sequenced genetic elements such as promoters (P), 5′ untranslated regions (U), signal peptides (SP), coding sequences (CDS) and terminators (T). Transcription units are assembled from selected level 0 modules using a one-pot one-step cloning reaction. Multigene constructs are then assembled in a second cloning step (and optionally further steps) from the transcription units. (**B**) Level 0 modules of different classes are flanked by compatible fusion sites. Each fusion site consists of 4 nucleotides of choice (boxed) flanked by a type IIS enzyme recognition site on the left or right side (vertical box drawn under the fusion site). (**C**) Examples of assembled transcription units for secreted or cytosolic proteins. The transcription unit for the cytolic protein was assembled from 4 modules rather than 5, using a CDS module cloned between fusion sites AATG and GCTT.

We have now developed a general modular cloning strategy (MoClo) to allow the systematic assembly of complete eukaryotic transcription units and of multigene constructs from basic pre-made standardized modules (**Fig. 1A**). Five basic module types (level 0 modules) were defined that include promoters, 5′ untranslated regions, signal peptides, coding sequences, and terminators. To enable assembly with the Golden Gate technology, each level 0 module type is flanked by specific fusion sites (**Fig. 1B**). Fusion sites overlapping with coding sequences were chosen so as to minimize changes to encoded proteins: the fusion site at the start codon was chosen to be AATG, while the fusion site between the signal peptide and the coding sequence was chosen to be AGGT, with GGT encoding a glycine, which is a common amino acid in signal peptides at position -1 [17]. Since the four remaining fusion site sequences (GGAG, TACT, GCTT and CGCT) are all positioned in non-translated sequences, they were selected with the only requirements as to be unique and non-palindromic to guarantee efficient cloning. For cytosolic proteins, which do not contain a signal peptide, the coding sequence can be cloned as a module flanked by AATG and GCTT fusion sites (**Fig. 1C**); such module type replaces the two modules SP and CDS of secreted proteins in assembly reactions. Since all level 0 modules from the same type are flanked by identical fusion sites, they are freely interchangeable, allowing any desired transcription unit to be created by simply choosing the modules needed. An assembled transcription unit represents a module again, albeit one of a higher order (level 1 module), which can be directionally assembled into a multigene construct (level 2) (**Fig. 1A**).

### Module generation: the level 0 and level 1 modules

**Figure 2:**
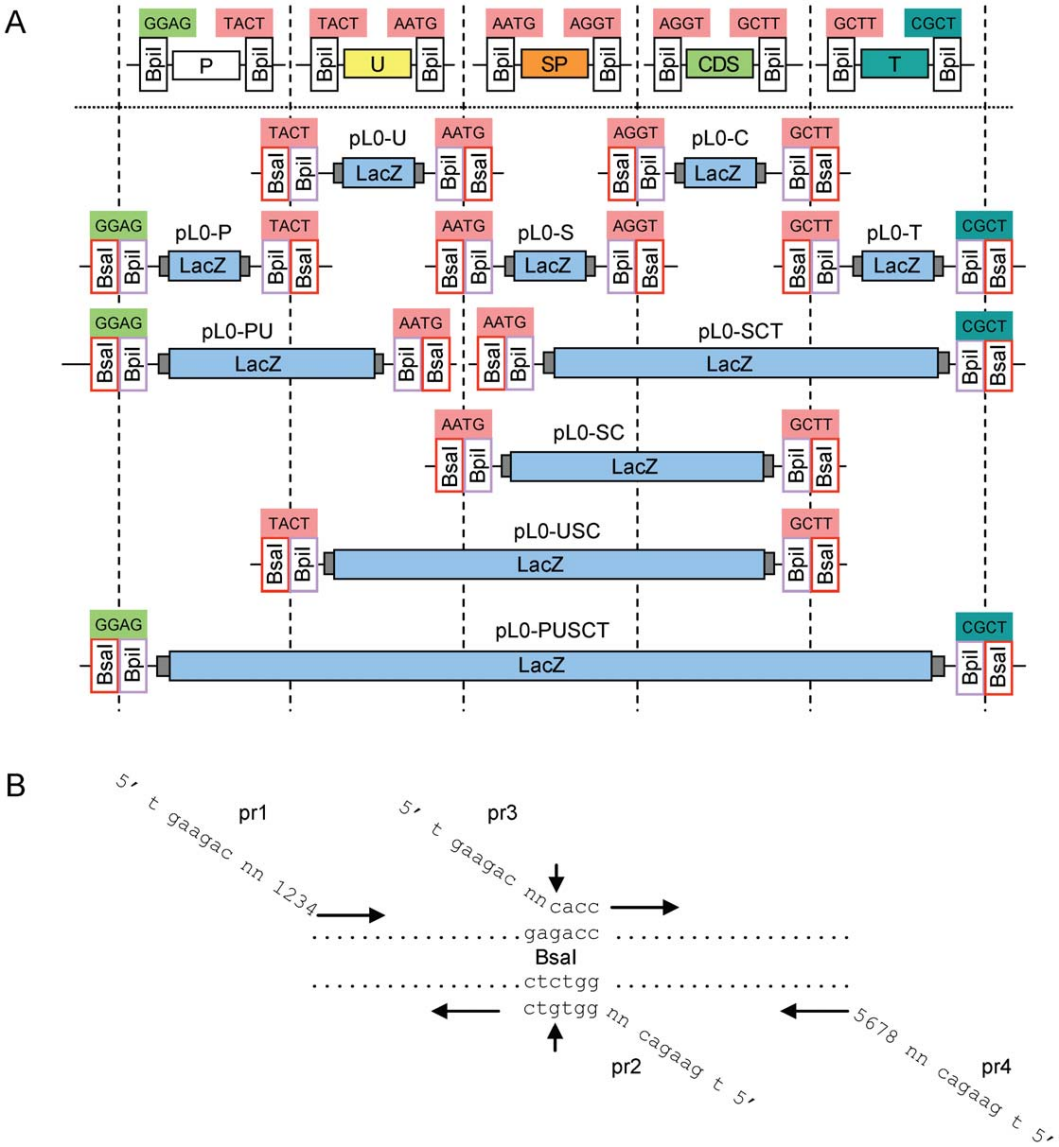
Level 0 destination vectors and principle for removal of internal sites from level 0 modules. (**A**) Level 0 destination vectors. Level 0 modules are made by amplification of selected sequences with primers adding flanking BpiI sites, and cloning of the amplified fragment (shown above the horizontal dotted line) via BpiI into the designated level 0 destination vectors (shown below). In addition to the 5 basic destination vectors, pL0-P, pL0-U, pL0-S, pL0-C and pL0-T, additional destination vectors allow cloning several genetic elements in one module. For example, plasmid pL0-SC can be used to clone sequences encoding cytosolic proteins, which do not contain a signal peptide. (**B**) Strategy for removing internal type IIS recognition sequences. Removal of a BsaI site in a fragment of interest is done by amplifying two fragments with primers pr1 and 2 and primers pr3 and 4. Primers pr2 and pr3 span the BsaI recognition site and introduce a single nucleotide mismatch (indicated by an arrow). As all primers have BpiI recognition sites in their 5′ extensions, the PCR fragments are cloned with a BpiI-based Golden Gate cloning reaction in the appropriate level 0 destination vector.

**Figure 3:**
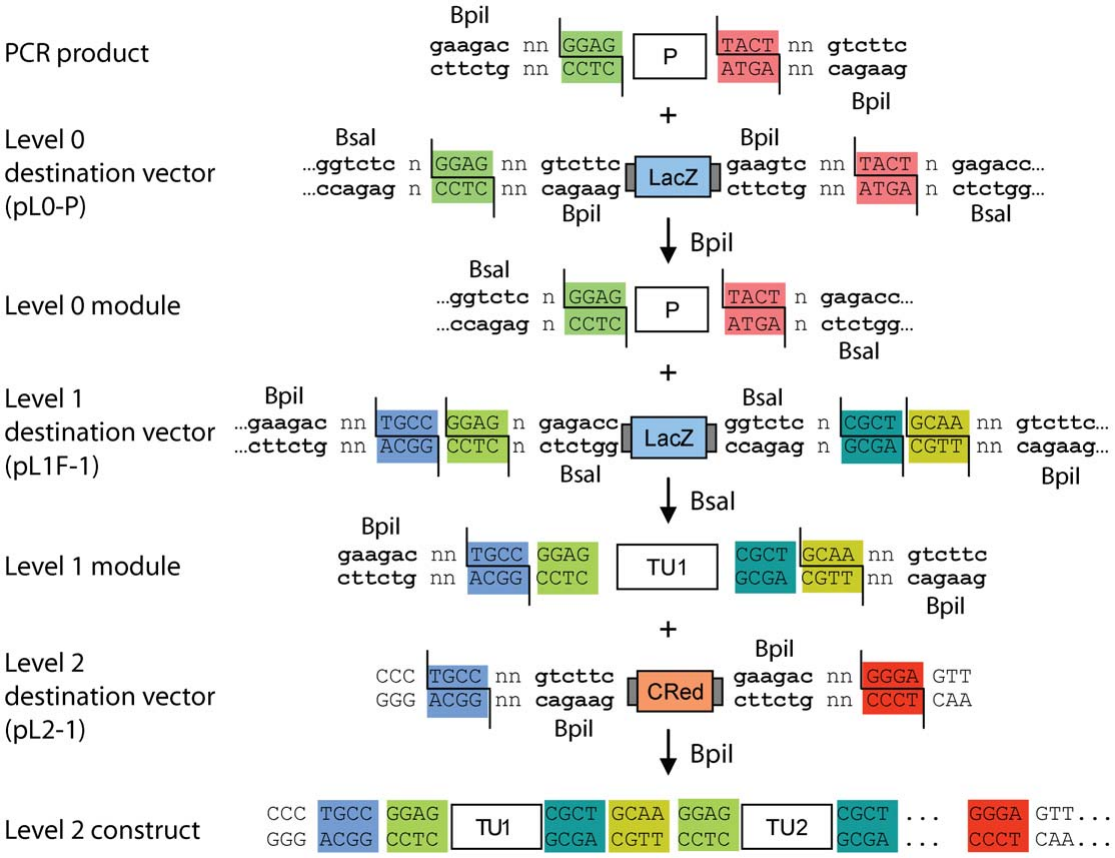
Arrangement of type IIS restriction sites and fusion sites for all assembly levels. A detailed overview of the organization and orientation of the type IIS restriction sites and the fusion sites at the different levels of the MoClo system is shown. A PCR product containing a promoter flanked by BpiI recognition sites and promoter-specific fusion sites (highlighted with color) is cloned via BpiI into the level 0 destination vector pL0-P. The promoter fragment in the resulting level 0 module is still flanked by the same fusion sites, but can now be released with BsaI. The level 0 promoter module and the other level 0 modules required to form a complete transcription unit (not shown) are then assembled via BsaI into a level 1 destination vector. As the fusion sites created by BsaI and BpiI do not overlap, the assembled level 1 module (here TU1) is equipped with two level 1-specific fusion sites (TGCC and GCAA). The level 1 module TU1 and other level 1 modules of choice (TU2 and not shown) can then be assembled via BpiI into the final level 2 construct in which no type IIS recognition sites are left. n indicates that any nucleotide can be used. CRed, red color selectable marker; P, promoter module; TU, assembled transcription unit.

In order to allow efficient cloning of the level 0 modules, a set of level 0 destination vectors was created (**Fig 2A**). Beside level 0 destination vectors for the five standard elements (pL0-P, pL0-U, pL0-S, pL0-C and pL0-T) further variants were included to provide the possibility to clone two or more genetic elements as a single module, for example promoter and 5′ untranslated region can be cloned as a single module using destination vector pL0-PU (**Fig. 2A**). Also, for cytosolic proteins that do not have a signal peptide, the coding sequence is cloned in vector pL0-SC rather than in vector pL0-C. All level 0 destination vectors are based on a pUC19 backbone, confer spectinomycin resistance (Sp^R^) and encode a *lacZα* fragment for blue/white selection. On both sides of the *lacZα* fragment two different type IIS recognition sequences - here BsaI and BpiI - are positioned in inverse orientation relative to each other, but creating the identical fusion site as exemplified by plasmid pL0-P in **figure 3**. This design allows cloning of the DNA fragment of interest efficiently via BpiI - removing the BpiI recognition sites and *lacZα* in the process - but provides the possibility to release the cloned fragment with BsaI creating the identical fusion sites it was cloned in. For cloning of level 0 modules, the designated sequences are PCR-amplified, adding the respective fusion site and a BpiI recognition site as part of the primers used for amplification, and cloned via a BpiI Golden Gate cloning reaction. Any internal type IIS recognition site for enzymes used in the MoClo system (BsaI, BpiI and later Esp3I) can be removed from the cloned fragment during this step by using primers overlapping but containing a single silent nucleotide mismatch in the recognition site (**Fig. 2B**).

**Figure 4:**
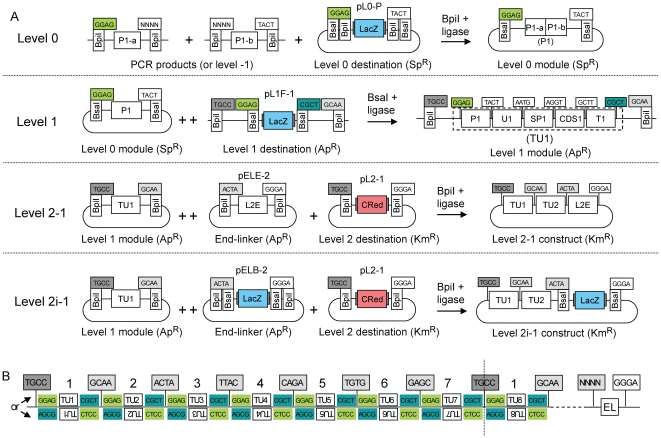
Modular cloning strategy. (**A**) Constructs are assembled by mixing in one tube all module plasmids (or PCR fragments for level 0) and a destination vector together with the appropriate type IIS enzyme (indicated above the arrows) and ligase. ++ indicates that only one of several modules was drawn due to space limitation. Each fusion site is shown as a box indicating its 4 nucleotides; the two boxes below show which type IIS recognition sites flank the fusion sites on the left and/or right sides. P1-a/b, promoter fragment a or b; U, 5′ untranslated region; SP, signal peptide; CDS, coding sequence; T, terminator; CRed, red color selectable marker; LacZ, *lacZα* fragment, blue color selectable marker; L2E, end-linker 2; Ap^R^, ampicillin resistance; Km^R^, kanamycin resistance; Sp^R^, spectinomycin resistance. (**B**) General structure of level 2 constructs. Transcription units are located between the sequences GGAG and CGCT (remnants of fusion sites used for assembly of transcription units in forward orientation) or AGCG and CTCC (for transcription units cloned in reverse orientation). The number above the transcription units indicates the relative position of the transcription units in the final construct (indicates which of the 7 level 1 destination vectors shown in figure 5 was used for assembly of this transcription unit). The construct is terminated at the right end by an end-linker (EL) that joins the downstream fusion site of the last transcription unit (NNNN) with the downstream fusion site of the destination vector (GGGA).

**Figure 5:**
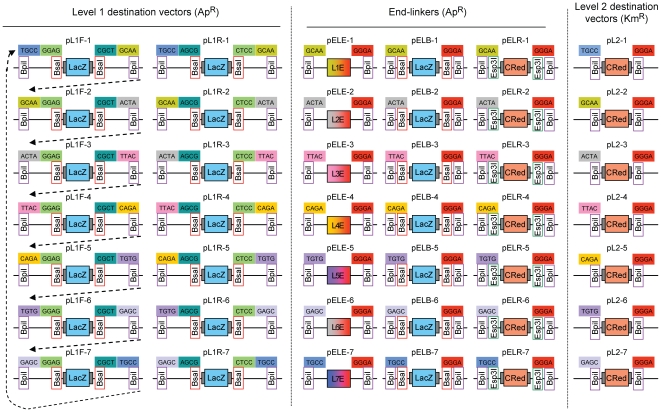
Vector set required for the MoClo system. All level 1 destination vectors (forward and reverse), level 2 destination vectors and the different end-linker sets are shown. Dotted arrows indicate the linear relationships between fusion sites in level 1 destination vectors. Compatible fusion sites are labeled with the same color.

Compatible sets of sequenced level 0 modules (for example promoter, 5′ untranslated region, signal peptide, CDS and terminator) are then assembled into a level 1 destination vector with a second Golden Gate reaction using the enzyme BsaI, leading to creation of a level 1 module, which contains a eukaryotic transcription unit (TU1, **Fig. 3** **and** **4A**). In contrast to the level 0 modules, the level 1 destination vectors confer ampicillin resistance, allowing efficient counter selection against level 0 module backbones. Similar to the level 0 destination vectors, a *lacZα* fragment is flanked on each side by two different type IIS recognition sites; however, here, the fusion sites defined by the two type IIS restriction enzymes are not identical (**Fig. 3** **and** **4A**). If, as for level 0 destination vectors, the cleavage sites of the two different type IIS enzymes (BsaI and BpiI) overlapped, all level 1 modules would be flanked by the same GGAG and CGCT fusion sites, making further directional cloning impossible. Therefore a series of 7 level 1 destination vectors was designed in which the BpiI restriction sites generate two fusion sites with new specificities for each plasmid (for example sites TGCC and GCAA for level 1 vector position 1, pL1F-1, **Fig. 3** **,** **4A** **and** **5**). These sites are compatible from one vector to the next so that multiple level 1 modules can be (again) directionally cloned into a level 2 destination vector. However, to avoid the construction and consideration of too many level 1 destination vectors, the spatial order of overhangs was designed to be circular instead of linear, as the first fusion site at position 1 (TGCC) is also the final site at position 7. So a level 1 module for position 1 can be reused later at a virtual position “8” (**Fig. 4B**). Due to this design, a maximum of 6 transcription units can be cloned in one step. A second set of level 1 destination vectors (pL1R-1 to 7, **Fig. 5**) was also created for cloning of transcription units in the reverse orientation using the same sets of level 0 modules. The combination of the two sets allow cloning of transcription units in either orientation at any position in level 2 constructs (**Fig. 4B**), giving the experimenter maximum freedom of design.

### Design of multigene constructs: level 2 and end-linkers

To provide flexibility in the design of multigene level 2 constructs, a set of seven level 2 destination vectors was made (**Fig. 5**). All level 2 destination vectors confer resistance to kanamycin and encode a red color selectable marker (CRed, containing an artificial bacterial operon responsible for canthaxanthin biosynthesis; see **Material and Methods**) which is flanked by two BpiI sites. The upstream fusion site of each level 2 destination vector is compatible with the upstream site of a corresponding level 1 module (for example TGCC in pL2-1). This design reduces the need for extensive recloning of the same transcription unit for different positions. For example, a level 1 module made for position 3 can easily be shifted to the relative first position when the level 2 destination vector pL2-3 is used, virtually deleting positions 1 and 2 (**Fig. 5**). The downstream fusion site, however, is unique to level 2 destination vectors (GGGA). The connection of the GGGA fusion site with the fusion site of the last assembled transcription unit in the DNA fragment is then realized by a set of end-linkers (pELE-n) (**Fig. 5**). Like the level 1 modules, the end-linker plasmids confer ampicillin resistance, and the end-linkers are flanked by BpiI sites. The desired multigene level 2 constructs are then assembled with BpiI from the chosen level 1 modules, a matching end-linker and a level 2 destination vector (**Fig. 3** **and** **4A**).

**Figure 6:**
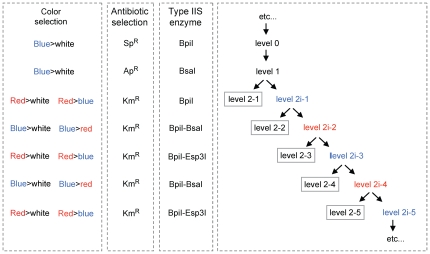
The MoClo cloning principle can be repeated indefinitely. Every cloning step relies on three elements that are different from one level to the next: antibiotic selectable marker, type IIS enzyme(s), and color selectable marker. Cloning after level 2i-1 requires the simultaneous use of two type IIS enzymes: BpiI/BsaI or BpiI/Esp3I.

The use of a basic end-linker (pELE-n), however, limits the maximal number of transcription units that can be cloned in a level 2 construct to six, because no type IIS restriction sites are left in the final construct, preventing further rounds of cloning (Level 2-1 construct; **Fig. 4A**). To provide an option for the addition of more transcription units, two additional end-linker sets were designed. These sets, pELB-n and pELR-n, provide two new type IIS recognition sites (BsaI for pELB-n and Esp3I for pELR-n) and a color selectable marker (*lacZα* fragment for pELB-n and CRed for pELR-n) to the assembled construct. The use of a pELB-n end-linker rather than a basic end-linker in the first round of assembly results into a level 2i (intermediate) construct (Level 2i-1; **Fig. 4A**), which contains, in addition to the cloned transcription units, two BsaI restriction sites for a next round of cloning and a *lacZα* fragment as selectable marker. The use of a pELR-n end-linker at the next step would lead to a level 2i-2 construct containing two Esp3I restriction sites and CRed selectable marker. The alternate use of end-linkers from the two sets pELB-n and pELR-n for successive cloning steps allows the process to be repeated indefinitely from the stand point of the cloning strategy (**Fig. 6**), but will ultimately be limited by construct size for transformation in standard bacterial hosts such as *E. coli*.

### Assembly of 11 transcription units in three steps

To test the system, we cloned a number of level 0 modules, removing at the same time all internal interfering type IIS recognition sites from the cloned sequences. These include 11 ORFs representing a wide spectrum of biological functions like immunoglobulins (IgG_1_ heavy and light chain), structural viral proteins from BTV (Blue tongue virus) and PVX (Potato virus X), the silencing inhibitor p19, the bar resistance marker and GFP. Since the number of commonly used promoters and terminator sequences available for expression of heterologous proteins in plants is low (our laboratory uses plants as expression host), and to avoid repetitive sequences in the planned multigene construct, we also cloned several *Arabidopsis thaliana* promoter and terminator sequences from genes which show a high expression level in leaves [18]. A summary of all level 0 modules used in this study is presented in **Table S1**.

#### Step one: Construction of level 1 modules

**Figure 7:**
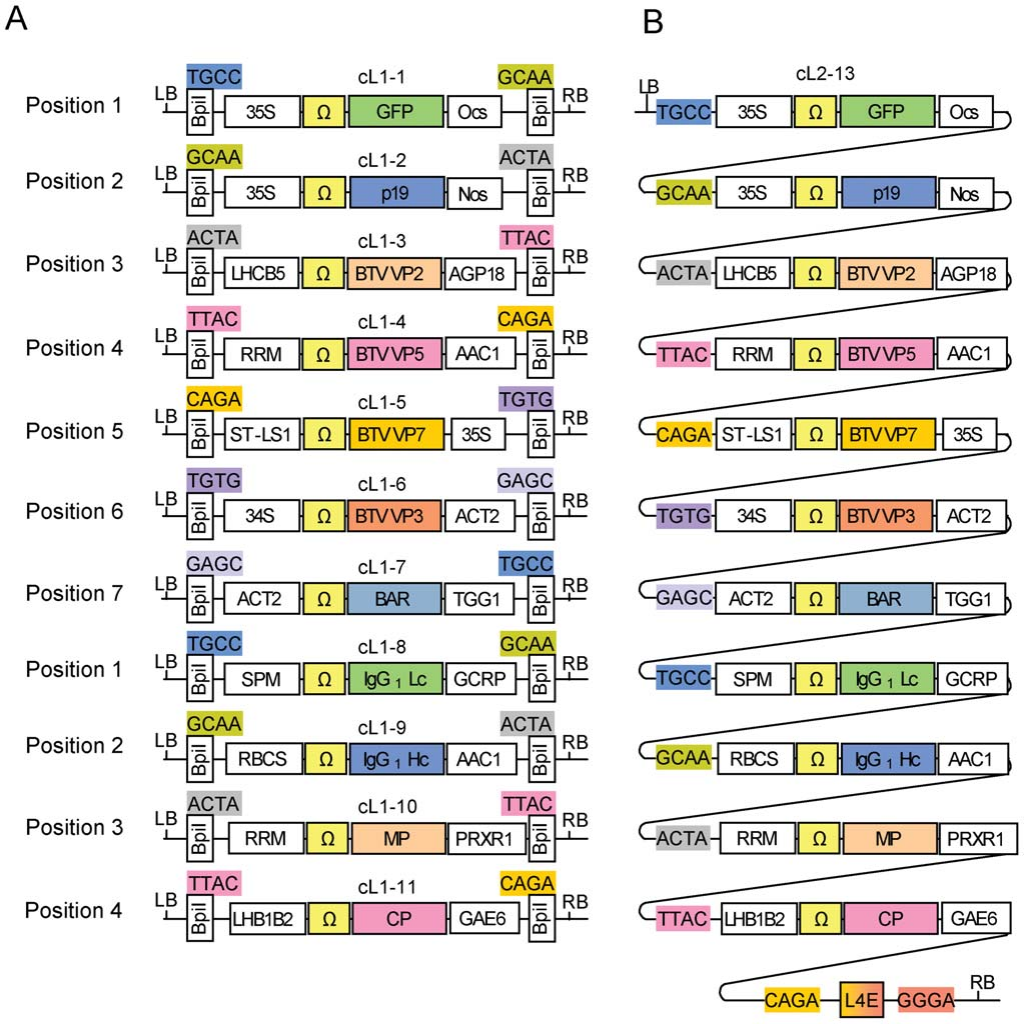
Structure of the eleven level 1 modules (A) and the final level 2 construct cL2-13 (B). All transcription units were assembled from 5 plasmids: 4 level 0 modules (promoter, 5′ untranslated region, CDS, and terminator) and a destination vector. All proteins are cytosolic except the two from constructs cL1-8 and 9 which are secreted. For both of these, the coding sequence module already contained the signal peptide. LB, T-DNA left border; RB, T-DNA right border; Ω, tobacco mosaic virus 5′ untranslated region; genetic elements used are listed in **Table S1**.

**Figure 8:**
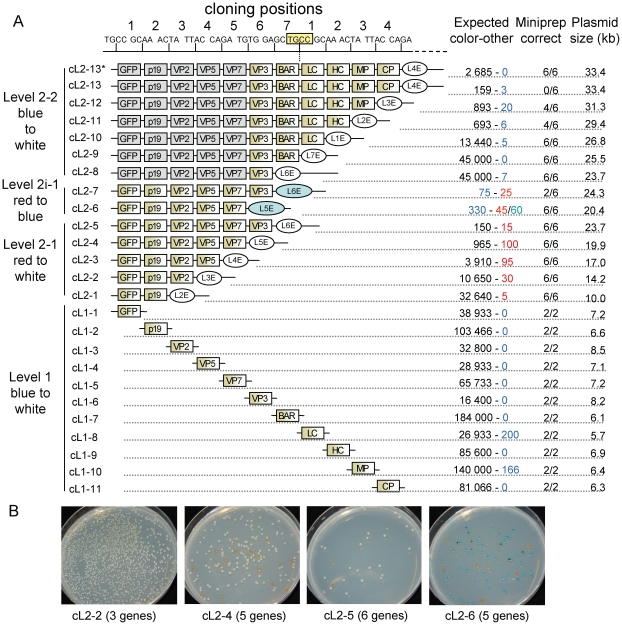
Cloning efficiency of level 1 and 2 constructs. (**A**) Assembled transcription units are schematically represented as boxes annotated with the name of the CDS they contain. Transcription units shown in grey were cloned in the previous step (in construct cL2-6). The respective cloning position of each transcription unit is indicated on the top. For level 2 constructs, the end-linker is shown as a circle. The number of colonies obtained per transformation is shown by color type, with the first number corresponding to the expected correct constructs (for cL2-6, wrong clones could be either red or green). (**B**) Plates from transformation of constructs for level 2-1 (cL2-2, cL2-4 and cL2-5) and level 2i-1 (cL2-6). Since level 2-1 cloning uses red/white selection, the correct constructs should be white, while colonies containing the original destination vector construct should be red. Level 2i-1 uses a blue/red selection, with colonies containing correct constructs expected to be blue, whereas incorrect ones can be red or green (contain both the canthaxanthin operon and the *lacZα* fragment).

As a first step towards a construct encoding 11 transcription units, the level 0 modules were assembled into 11 artificial transcription units. Promoters and terminators were randomly assigned to ORFs without consideration for potential level of expression, since all constructs described next were made purely as an exercise to demonstrate the ability of the MoClo system to clone multigene constructs. The designated transcription units were also randomly assigned to one of the seven level 1 positions (**Fig. 7A**). In 11 independent cloning reactions, the level 0 modules were combined with the respective level 1 destination vectors, T4 DNA ligase and the restriction enzyme BsaI in a one-tube one-step reaction. The different antibiotic resistances of level 0 and level 1 destination vectors used in combination with the blue/white selection provide a convenient way to screen for correctly assembled level 1 modules. After transformation, the reactions were spread on plates containing ampicillin and X-Gal and the numbers of white and blue colonies were counted. The number of white colonies (expected for the correct constructs) varied from approximately 16,000 to 180,000, whereas a few blue colonies (<1%) were present in only two out of eleven reactions (constructs level 1 cL1-1 to cL1-11; **Fig. 8A**). Plasmid DNA from two white colonies from each reaction were analyzed by an analytical endonuclease cleavage with BpiI (which cleaves on both sides of the assembled transcription unit). All 22 tested plasmids contained a fragment of the expected size (not shown).

#### Step two: Assembly of up to six level 1 modules into a level 2 construct

As a next step, we analyzed how efficiently multiple level 1 modules could be assembled into a level 2 construct. Therefore, five BpiI-based Golden Gate cloning reactions were set up, including two to six level 1 modules, the appropriate end-linker (pELE-2 to pELE-6) and the level 2 destination vector (pL2-1) (constructs level 2 cL2-1 to cL2-5**,** **Fig. 8A**). The kanamycin resistance and the CRed color selection marker of the level 2 destination vector permits an effective counter-selection against the level 1 module plasmids and a red/white color selection for correctly assembled level 2 constructs. The number of white colonies obtained per transformation, which is a measure of cloning efficiency, decreased from approximately 33,000 (for two level 1 modules plus end-linker) to 150 (six level 1 modules plus end-linker), and the percentage of incorrect red colonies increased from 0.02% to 10% (**Fig. 8A and B**). Six white colonies were analyzed from each level 2 construct assembly by analytical endonuclease cleavage of plasmid DNA and all were correct (not shown).

As shown above, the assembly of a 24 kb construct (cL2-5) encoding six transcription units can be done in a single one-step and one-tube reaction from level 1 modules. However the final level 2 constructs are in a “closed” status as no type IIS restriction sites are left, prohibiting the insertion of additional genes. In order to extend the number of transcription units beyond six, new type IIS recognition sites have to be incorporated into the level 2 constructs. Therefore constructs cL2-4 and cL2-5 were recreated, but using end-linkers pELB-5 and pELB-6 instead of pELE-5 and pELE-6. These new end-linkers provide two new BsaI restriction sites and a *lacZα* fragment to the final constructs cL2-6 and cL2-7. In contrast to the red/white selection used for pL2-4 and pL2-5, red/blue selection is used. In addition to correct blue and incorrect red colonies, dark green colonies were also obtained; these contain incorrect plasmids with both the CRed operon and a *lacZα* fragment (**Fig. 7B**). Although the efficiency dropped for the last construct, correct constructs were obtained for both reactions. The correctly assembled constructs were used for a further round of assembly.

#### 3^rd^ step: Assembly of the final 33 kb construct

Level 2i-1 plasmid cL2-6 was chosen as a destination vector for the introduction of up to six additional transcription units. In contrast to the previously described assembly steps, two type IIS restriction enzymes have to be used in the same mix. BsaI reopens the level 2i-1 backbone and provides defined fusion sites which are compatible with the level 1 modules released by BpiI. Since two type IIS restriction enzymes have to be used at the same time and since the level 2i-1 destination vector cL2-6 has already a size of 20 kb, we tested again the efficiency of the Golden Gate cloning. One to six additional modules were assembled with the appropriate end-linkers resulting in constructs cL2-8 to cL2-13. The cloning efficiency decreased with increasing number of incorporated modules (**Fig. 8A**). Interestingly, the rate with which the cloning efficiency drops is similar to the earlier analyzed set of level 2-1 plasmids (cL2-1 to cL2-5) despite the different destination vector size of 22 kb versus 4 kb. In case of the largest construct (cL2-13), no positive clone was identified. The cloning reaction was repeated using different Golden Gate cloning conditions with a program providing alternating cycles optimal for restriction and ligation. These conditions increased the total number of white colonies, and all six tested cL2-13* constructs were correct (the final construct is shown in **Fig. 7B**).

**Figure 9:**
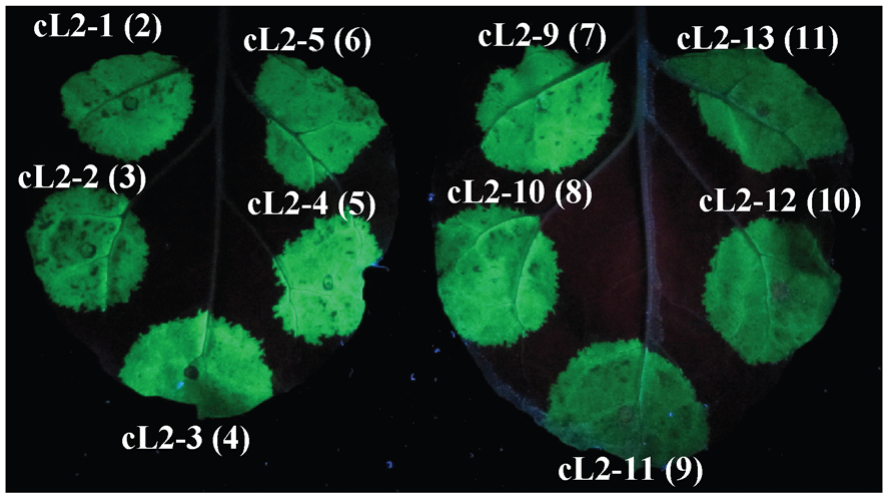
Expression of GFP by level 2 constructs. Level 2 constructs in *Agrobacterium tumefaciens* were inoculated into *N. benthamiana* leaves. GFP expression was observed at 5 dpi under UV light. The number in parenthesis indicates the number of transcription units in each infiltrated construct.

To verify that the transcription units assembled with this system are functional, we tested expression of GFP from all level 2 constructs (pL2-1 to pL2-13). All were introduced into *Agrobacterium tumefaciens* and inoculated into *Nicotiana benthamiana.* As expected, GFP under control of a 35S promoter is expressed for all constructs (**Fig. 9**).

## Discussion

We have shown here that complex constructs containing many transcription units (here 11, consisting of 44 individual basic modules) can be assembled by a series of three one-pot Golden Gate cloning reactions. The construction principle exemplified in this work can theoretically be repeated indefinitely to add more transcription units, until the constructs become simply too large to be transformed or propagated in standard hosts such as *E. coli*. As outlined in figure 6, it is necessary to create a destination vector at each level 2 cloning step for further rounds of cloning (level 2i-2 to level 2i-3, etc…). This is done by the alternating use of end-linkers providing different type IIS restriction sites (for example Esp3I or BsaI) and allowing convenient color selection from blue to red and vice versa (**Fig. 6**). The expansion, for example, of the largest construct made in this study (cL2-13, level 2-2, 33 kb) would require its reconstruction, but with an end-linker that adds two Esp3I restriction sites to the construct (end-linker pELR-4, **Fig. 5**). One or more genes could then be added to this level 2i-2 destination vector using an Esp3I/BpiI Golden Gate cloning reaction (**Fig. 6**).

Beside the construction of large and complex constructs encoding entire pathways, the high cloning efficiency also allows the creation of construct libraries. Instead of using one specific module for each component of a transcription unit, a module library can be used instead. In case a library of promoters is used, constructs obtained would contain a coding sequence under control of different promoters. Since nearly all constructs are correct, the library can be screened directly for optimal expression level for this particular gene, or be used for the next level of cloning in which several genes or again gene libraries are assembled. This application is of particular interest for the optimization of biochemical pathways for metabolic engineering where several genes not only have to be co-expressed, but also, their expression ratios have to be balanced to obtain optimal yield of the desired product.

The advantages of using standardized modules do not lie exclusively in the ability to easily create complex constructs. Already, the simple definition of a general cloning standard will result in tremendous synergistic effects, since the validated modules or module libraries created by different scientific groups can be reused from the whole scientific community. An impressive example is the widely used standard proposed by the BioBrick foundation [10], [19]. Here, researchers from all over the world have already contributed thousands of compatible modules to a freely available module collection. In contrast to MoClo, Biobrick modules are flanked by standard type II restriction enzymes, and assembly of two BioBricks via restriction and ligation results in an idempotent new Biobrick module. However, the two modules are separated by a scar sequence, and the process is unsuitable for the assembly of multiple fragments in one step.

The principle that a huge community contributes to a standardized system requires however that the standard shows some flexibility. Although the MoClo system described here is based on five basic modules, it is very versatile since each of these modules can be subdivided in smaller modules that would still be compatible with the existing ones. For example, a terminator can be split in two modules consisting of 3′ untranslated region and actual terminator sequences by definition of a new fusion site separating both modules. The transcription unit would be assembled with the two new modules replacing the original terminator. In case of more sophisticated cloning applications, like the shuffling of an ORF consisting of several protein modules, it may be favorable to define an entire new level. These level -1 modules have to follow the same principles as all other modules: a set of compatible overhangs, where the first and the last are compatible to the next level, a specific color selection and a specific antibiotic selection marker have to be defined.

The data presented here show that all elements required for the design of a completely automated cloning system are now in place. Operations that are required for cloning using the MoClo system consist of preparation of plasmid DNA, liquid handling and incubation to perform restriction-ligation, plating of transformation on plates, picking of colonies, and digestion and analysis of plasmid DNA. The last step can even be replaced by DNA sequencing of a single colony, because the system is so efficient. A further advantage in terms of automation is that no sophisticated construction strategies are needed since the design is automatically defined by the number and the order of modules that a user wants to assemble. The cloning strategy can be easily and unambiguously determined by a simple computer program, which could also be directly linked to the automation robots that would make the construct.

## Materials and Methods

### Molecular biology reagents

Restriction enzymes used in this study were purchased from New England Biolabs (Frankfurt, Germany) and Fermentas (St. Leon-Rot, Germany). T4 DNA ligase was purchased from Promega (Mannheim, Germany). Plasmid DNA preparations were made by using the NucleoSpin Plasmid Quick Pure kit (Macherey-Nagel, Dueren, Germany) following the manufacturer protocol. Plasmid DNA concentration was measured using a Nano Drop® Spectrophotometer ND-2000 (Peqlab, Erlangen, Germany).

### MoClo cloning protocol

Restriction-ligations were set up by pipetting in one tube approximately 40 fmol (∼100 ng of DNA for a 4 kb plasmid) of each DNA component (PCR product or plasmid), 10 U of the required restriction enzyme (BsaI or BpiI) and 10 U T4 DNA ligase (using high concentration ligase, 20 U/µl) in Promega ligation buffer in a final reaction volume of 20 µl. The reaction was incubated in a thermocycler for 5 hours at 37°C, 5 min at 50°C and 10 min at 80°C. The reaction mix was then added to 100 µl chemically competent DH10b cells, incubated for 15–30 min on ice and transformed by heat shock. 800 µl of liquid LB was then added to the transformation, and the cells were let to recover 45 min at 37°C. Different aliquots of the transformation were plated on LB plates containing the appropriate antibiotic. The number of colonies was counted for one or two selected plates (containing countable number of colonies), or from a section of the plates when very high number of colonies were obtained even for the lowest volume plated. The number of colonies was then extrapolated for the entire transformation.

For level 2-2 cloning, two type IIS enzymes were required, BpiI and BsaI. The same protocol was used as described above except that 10 U and 2.5 U were used for the enzymes BpiI and BsaI, respectively. To optimize efficiency of the restriction-ligation for the final construct containing 11 transcription units (cL2-13*), a variation of this protocol was used as follows. The reaction mix was set up containing 20 U ligase, 5 U BpiI and 5 U BsaI, in a total reaction volume of 20 µl. The mix was incubated in a thermocycler with the following parameters: incubation for 2 minutes at 37°C, 5 minutes at 16°C, both steps repeated 45 times, followed by incubation for 5 minutes at 50°C and 10 minutes at 80°C. The reaction mix was transformed in *E. coli* chemically competent cells as described above.

### Cloning of the canthaxanthin biosynthesis operon

A DNA fragment containing genes for canthaxanthin biosynthesis was made by PCR amplification of 4 genes from *Pantoea ananatis* that are necessary for biosynthesis of β-carotene (genes *crtE*, *crtY*, *crtI* and *crtB*) [20] and of one gene from *Agrobacterium aurantiacum* (*crtW*) necessary to convert β-carotene to canthaxanthin [21]. c*rtW* is used in addition to the 4 Pantoea genes because the orange/red color of canthaxanthin is more visible on agar plates than the yellow color of β-carotene. The *Pantoea ananatis* strain was obtained from the DSMZ (cat. DSM 30080), and a fragment containing *crtW* was synthesized by Mr. Gene GmbH (Regensburg, Germany). An artificial operon containing *crtE-W-Y-I-B* under control of the *P. ananatis* native promoter was made by ligation of three fragments derived from PCR: fragment 1 containing the promoter and *crtE* was amplified from *P. ananatis* genomic DNA with primers 5′-ttt ggtctc a ggag ggtaccgcacggtctgccaa and 5′-ttt ggtctc a tcatgcagcatccttaactgacggcag, fragment 2 containing *crtW* was amplified from a synthetic DNA fragment (sequence identical to the native sequence) with primers 5′-ttt ggtctc a atgagcgcacatgccctgcc and 5′-ttt ggtctc a tcactcatgcggtgtcccccttggt, and fragment 3 containing *crtY-I-B* was amplified from *P. ananatis* DNA using primers 5′-ttt ggtctc a gtgacttaagtgggagcggctatg and 5′-ttt ggtctc a atgtagtcgctctttaacgatgag. The fragments were assembled by Golden Gate cloning in a target vector using BsaI. Two BpiI and one Esp3I site present in *crtY* were removed using primers containing silent mutations in the recognition sites.

## Supporting Information

Table S1 Level 0 modules used in this study.
(DOC)
