# Supplementary material for: A Modular Cloning System for Standardized Assembly of Multigene Constructs
Source: PLoS One. 2011 Feb 18;6(2):e16765. doi: 10.1371/journal.pone.0016765 (PMC3041749; doi:10.1371/journal.pone.0016765)
Supplement: Table S1 — Level 0 modules used in this study. (DOC) [file pone.0016765.s001.doc]

**Table S1: Level 0 modules used in this study**

| **Module type and construct number** | Relevant characteristics | **Reference or accession number** |
| --- | --- | --- |
| **Promoter (P)** | | |
| pICH41373 | 35S promoter | *CaMV* [1] |
| pICH41551 | ST-LS1 (Stem and leaf specific) promoter | *S. tuberosum*; [2] |
| pICH42755 | 34S promoter | *FMV* [3] |
| pICH42760 | Spm promoter | *Zea mays* [4] |
| pICH44157 | RBCS (RuBisCO Small subunit 1b) promoter | *A. thaliana*; [At5g38430](http://www.ncbi.nlm.nih.gov/sites/entrez?Db=gene&Cmd=retrieve&dopt=full_report&list_uids=833830&log$=databasead&logdbfrom=nuccore); This work |
| pICH45131 | LHB1B2 promoter | *A. thaliana*; At2g34420; This work |
| pICH45145 | LHCB5 promoter | *A. thaliana*; At4g10340; This work |
| pICH45167 | RRM-containing protein promoter | *A. thaliana*; At1g70200; This work |
| pICH50581 | ACT2 (Actin 2) promoter | *A. thaliana*; [At3g18780](http://www.ncbi.nlm.nih.gov/entrez/utils/fref.fcgi?http://www.tigr.org/tigr-scripts/euk_manatee/shared/ORF_infopage.cgi?db=ath1&orf=AT3G18780); This work |
|  |  |  |
| **5’UTR (U)** | | |
| pICH46501 | Tabacco mosaic virus Ω fragment | This work |
|  |  |  |
| **CDS** | | |
| pICH41531 | sGFP codon optimized | [5] |
| pICH42222 | Basta™ resistance protein  (Phosphinothricin acetyltransferase) | *S. hygroscopicus* [6] |
| pICH44022 | P19 *Tomato bushy stunt virus* silencing inhibitor | [7] |
| pICH45502 | BTV (*blue tongue virus*) VP2 | This work |
| pICH45512 | BTV (*blue tongue virus*) VP3 | This work |
| pICH45526 | BTV (*blue tongue virus*) VP5 | This work |
| pICH45531 | BTV (*blue tongue virus*) VP7 | This work |
| pICH48348 | PVX CP | This work |
| pICH48367 | PVX 25K MP | This work |
| pICH49488 | IgG_1_ Light chain with native signal peptide | This work |
| pICH49500 | IgG_1_ Heavy chain with native signal peptide | This work |
|  |  |  |
| **Terminator (T)** | | |
| pICH41432 | Ocs terminator | *A. tumefaciens* [8] |
| pICH41414 | 35S terminator | *CaMV* |
| pICH44300 | ACT2 (Actin 2) terminator | *A. thaliana*; [At3g18780](http://www.ncbi.nlm.nih.gov/entrez/utils/fref.fcgi?http://www.tigr.org/tigr-scripts/euk_manatee/shared/ORF_infopage.cgi?db=ath1&orf=AT3G18780); This work |
| pICH44311 | TGG1 (Thioglucoside Glucohydrolase 1) terminator | *A. thaliana*; At5g26000; This work |
| pICH44344 | GCRP (glycine-rich protein) terminator | *A. thaliana*; [At1g67870](http://www.arabidopsis.org/servlets/TairObject?type=locus&name=AT1G67870); This work |
| pICH44355 | AAC1 (ADP/ATP carrier protein 1) terminator | *A. thaliana*; At3g08580; This work |
| pICH44377 | PRXR1 (Peroxidase 1) terminator | *A. thaliana*; [At4g21960](http://www.ncbi.nlm.nih.gov/entrez/utils/fref.fcgi?http://www.tigr.org/tigr-scripts/euk_manatee/shared/ORF_infopage.cgi?db=ath1&orf=AT4G21960); This work |
| pICH44388 | AGP18 (Arabinogalactan protein 18) terminator | *A. thaliana*; [At4g37450](http://www.ncbi.nlm.nih.gov/entrez/utils/fref.fcgi?http://www.tigr.org/tigr-scripts/euk_manatee/shared/ORF_infopage.cgi?db=ath1&orf=AT4G37450); This work |
| pICH44393 | GAE6 (UDP-D-Glucoronate 4-Epimerase 6) terminator | *A. thaliana*; [At3g23820](http://www.arabidopsis.org/servlets/TairObject?type=locus&name=AT3G23820); This work |
| pICH49344 | Nos terminator | *A. tumefaciens* [9] |

1. Guilley H, Dudley RK, Jonard G, Balazs E, Richards KE (1982) Transcription of Cauliflower mosaic virus DNA: detection of promoter sequences, and characterization of transcripts. Cell 30: 763-773.

2. Stockhaus J, Eckes P, Blau A, Schell J, Willmitzer L (1987) Organ-specific and dosage-dependent expression of a leaf/stem specific gene from potato after tagging and transfer into potato and tobacco plants. Nucleic Acids Res 15: 3479-3491.

3. Sanger M, Daubert S, Goodman RM (1990) Characteristics of a strong promoter from figwort mosaic virus: comparison with the analogous 35S promoter from cauliflower mosaic virus and the regulated mannopine synthase promoter. Plant Mol Biol 14: 433-443.

4. Raina R, Cook D, Fedoroff N (1993) Maize Spm transposable element has an enhancer-insensitive promoter. Proc Natl Acad Sci U S A 90: 6355-6359.

5. Chiu W, Niwa Y, Zeng W, Hirano T, Kobayashi H, et al. (1996) Engineered GFP as a vital reporter in plants. Curr Biol 6: 325-330.

6. Thompson CJ, Movva NR, Tizard R, Crameri R, Davies JE, et al. (1987) Characterization of the herbicide-resistance gene bar from Streptomyces hygroscopicus. Embo J 6: 2519-2523.

7. Marillonnet S, Thoeringer C, Kandzia R, Klimyuk V, Gleba Y (2005) Systemic Agrobacterium tumefaciens-mediated transfection of viral replicons for efficient transient expression in plants. Nat Biotechnol 23: 718-723.

8. De Greve H, Dhaese P, Seurinck J, Lemmers M, Van Montagu M, et al. (1982) Nucleotide sequence and transcript map of the Agrobacterium tumefaciens Ti plasmid-encoded octopine synthase gene. J Mol Appl Genet 1: 499-511.

9. Depicker A, Stachel S, Dhaese P, Zambryski P, Goodman HM (1982) Nopaline synthase: transcript mapping and DNA sequence. J Mol Appl Genet 1: 561-573.
